# A Combined Magnetoelectric Sensor Array and MRI-Based Human Head Model for Biomagnetic FEM Simulation and Sensor Crosstalk Analysis

**DOI:** 10.3390/s24041186

**Published:** 2024-02-11

**Authors:** Mesut-Ömür Özden, Giuseppe Barbieri, Martina Gerken

**Affiliations:** Integrated Systems and Photonics, Department of Electrical and Information Engineering, Kiel University, Kaiserstraße 2, 24143 Kiel, Germany; giba@tf.uni-kiel.de

**Keywords:** biomagnetic sensor, crosstalk, finite-element method (FEM), human head model, magnetic fields, magnetoelectric effect, MRI data, multiferroic device, multiscale model, sensor array

## Abstract

Magnetoelectric (ME) magnetic field sensors are novel sensing devices of great interest in the field of biomagnetic measurements. We investigate the influence of magnetic crosstalk and the linearity of the response of ME sensors in different array and excitation configurations. To achieve this aim, we introduce a combined multiscale 3D finite-element method (FEM) model consisting of an array of 15 ME sensors and an MRI-based human head model with three approximated compartments of biological tissues for skin, skull, and white matter. A linearized material model at the small-signal working point is assumed. We apply homogeneous magnetic fields and perform inhomogeneous magnetic field excitation for the ME sensors by placing an electric point dipole source inside the head. Our findings indicate significant magnetic crosstalk between adjacent sensors leading down to a 15.6% lower magnetic response at a close distance of 5 mm and an increasing sensor response with diminishing crosstalk effects at increasing distances up to 5 cm. The outermost sensors in the array exhibit significantly less crosstalk than the sensors located in the center of the array, and the vertically adjacent sensors exhibit a stronger crosstalk effect than the horizontally adjacent ones. Furthermore, we calculate the ratio between the electric and magnetic sensor responses as the sensitivity value and find near-constant sensitivities for each sensor, confirming a linear relationship despite magnetic crosstalk and the potential to simulate excitation sources and sensor responses independently.

## 1. Introduction

In the field of medical diagnostics, bioelectric measurements are commonly performed on patients to investigate possible pathological disorders. Well-known applications of this kind are the electrocardiogram (ECG) and the electroencephalogram (EEG), which measure and evaluate the electric activities of the human heart and brain, respectively. To perform them, electrodes are applied directly on the patient’s body and the electric potential generated by the heart or brain activity is measured on the surface of the skin. While electrical measurements such as ECG and EEG are of the utmost importance and allow physicians to gather valuable vital information on a patient’s health with cost-effective and widely available machines and with high temporal resolution, they do have drawbacks as well, leading to increasing interest in measurement applications based on the magnetic sensing of biological signals. As an alternative or complementary measurement technique to bioelectric signals [1], biomagnetic signals can also be measured with appropriate sensing devices such as fluxgate magnetometers [2,3], optically pumped magnetometers [4], SQUID systems [5], and magnetoelectric (ME) magnetic field sensors [6,7,8,9].

In current clinical and research applications, SQUID sensors are used as the gold standard for magnetic measurements down to the femtotesla (fT) range, enabling their use in applications such as magnetoencephalography (MEG) [5,10]. Multi-channel SQUID magnetometers with helmet-shaped dewars have the ability to cover several head regions and reduce measurement times simultaneously [5], and state-of-the-art devices achieve a noise spectral density of approximately 3 fT/Hz^1/2^ [11], allowing their efficient and precise application in MEG.

While SQUIDs are still the sensing device of choice for many applications due to their extremely high sensitivity, ME sensors were recently shown to be promising in highly versatile and novel applications ranging from automatic real-time magnetic localization of an ultrasound probe [12], to magnetic detection of positions and orientations of deep brain stimulation (DBS) electrodes in patients [13], to magnetic motion analysis for swallowing detection in individuals suffering from dysphagia [14]. Crucially, measurements in the operation room or even wearable solutions could be possible with ME sensors for applications such as the detection and orientation of DBS electrodes or swallowing detection, which are currently not feasible with SQUID systems.

Even outside the scope of biomagnetic sensing, ME devices have the potential for a variety of applications, such as energy-efficient memory [15,16], antennas and energy harvesting [17,18], electric current sensing [19], and automotive applications [20]. Lastly, as opposed to many other magnetic sensing systems, ME sensors also offer the potential for room-temperature, passive and unshielded operation [9,21]. In this work, we demonstrate a combined multiscale 3D finite-element method (FEM) model including several ME sensors in different array configurations and a detailed anatomical human head model based on MRI data. Previous investigations report simplified spherical or realistic anatomical head models and magnetic field calculations for specific applications such as the EEG or MEG forward problem. Extremely detailed models on EEG and MEG applications exist, which even consider how the movement of the brain inside the cerebrospinal fluid relative to the inner skull due to subjects’ changing body position can affect mesh generation [22,23]. However, the respective sensor systems for such applications are often not jointly evaluated at all, or simplified to point [24] or circular magnetometers [25]. However, ME sensors are not of a negligible size and their geometry plays a role in the sensitivity of measurements [26]. Therefore, the study framework presented in this work is necessary to simulate sensors and the head or throat in a single FEM model. In this case, we focus on the head, as the simulation framework is known from previous MEG cases, and we significantly extend this framework by integrating the sensors in the simulation. The key novelty of this approach is the complete integration of an MRI-based head model with a fully coupled ME sensor array model and its physical properties. This enables us to evaluate the mechanical, electrical, and magnetic behavior of the magnetoelectric sensing devices in a variable array configuration. Additionally, the inclusion of further components such as operation instruments could be added to the simulation framework.

With this extensive model, we aim to investigate the response of ME sensors to different excitation mechanisms such as homogeneously applied external magnetic fields and a dipole source inside the human head. We also evaluate the concept of magnetic crosstalk between adjacent ME sensors in the different array configurations, which is based on the high-permeability material utilized in the magnetostrictive layers of the devices. This crosstalk can lead to adverse sensing performance based on the location of each sensor inside the array, the distance to adjacent sensors and the excitation source, and the method of excitation (i.e., homogeneous vs. inhomogeneous excitation). Lastly, we calculate the ratio between electric sensor response and the magnetic flux density for each individual sensor in order to determine whether the separability of effects is visible in our combined FEM simulation, as expected for a linear model. While the inclusion of nonlinear effects due to magnetic material properties or secondary currents induced in the head requires a combined model, determining a simple linear relationship between the magnetic sensor excitation and the electric response justifies the investigation of excitation sources and sensor responses in separate models. This greatly reduces computational requirements, allows for higher resolution meshing, and enables arbitrary combinations of separately developed source and sensor models. 

**Figure 1:**
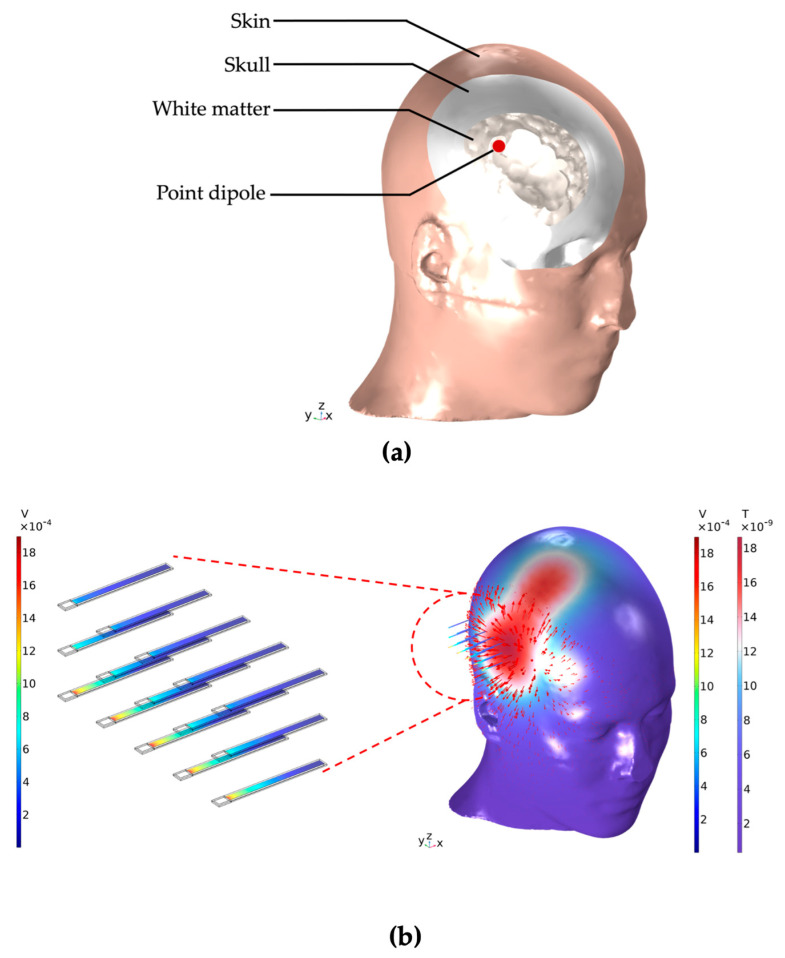
(**a**) Clipped view of the human head with its tissue regions of skin, skull, and white matter. The white matter region contains an electric current point dipole source—as would be obtained with a deep-brain stimulation electrode—which creates an electromagnetic field. This field propagates through the tissues of the head, through the air environment, and into the ME sensor array. Shown in (**b**) are the magnetic flux density norm on the head’s surface with corresponding vector arrows and, based on this propagating magnetic field as the method of excitation, the electric response of an adjacent ME sensor array with 15 sensors. The sensors are located at a distance of 1.5 cm from the head and a distance of 1 cm between neighboring sensors. The resulting absolute potential on the surface of each individual piezoelectric layer is shown.

Figure 1 visualizes the concept of the simulations presented in this work. In (a), we illustrate a clipped view of the head model with its tissue regions of skin, skull, and white matter, as well as the point dipole located in the latter region. (b) demonstrates an electric current point dipole source inside the head inducing the propagation of an inhomogeneous magnetic field. This magnetic field propagates through the head, into the surrounding air environment, and becomes the excitation source for the ME sensor array. The individual sensors within the array can display vastly different responses to this excitation based on the previously mentioned parameters, which we aim to systematically investigate and discuss throughout this work. 

Our study is divided into the following sections. Section 2 offers insights into the setup and geometries of the separate ME sensor and human head models and their merging into a joint multiscale 3D FEM model. We also describe our simulation method and variations within the model that are relevant to our investigation, as well as the process of obtaining the head model from raw MRI data. In Section 3, we showcase the results of magnetic field excitation and propagation, as well as the ME sensor array behavior for different excitation and array configurations. We highlight the magnetic and electric effects of the sensor response and investigate the magnetic crosstalk between adjacent sensors based on their position in the array and the relationship between electric response and magnetic excitation of the ME sensors. Lastly, Section 4 includes important discussion points of the obtained results and concludes our work with an emphasis on major insights gained by this study, as well as important implications for future research in the field of magnetoelectric sensors. 

## 2. Models and Methods

The MRI-based human head model, the single ME sensor model, and the ME sensor array model were developed and combined using COMSOL Multiphysics 6.1 with its built-in *solid mechanics*, *magnetic fields*, and *electrostatics* interfaces. The software was used to set up and perform 3D FEM simulations in the frequency domain with magnetic excitation at the cantilever sensor’s physical resonance frequency of 848 Hz. The software pipeline to process medical MRI data into the segmented 3D human head model, as well as the working principle, geometry, and physical properties of the ME sensors, will be explained in the following subsections. Lastly, we will describe the integration of both components into the combined multiscale head and sensor model before moving on to the results section of our study. We performed the simulations shown in this work using an Intel Xeon E5-2697A v4 CPU with 64 cores at 2.60 GHz, 503.8 GiB RAM and Ubuntu OS 22.04.3.

### 2.1. MRI-Based Human Head Model

In order to create an approximation of the head and improve the previous spherical model [27], MRI-based medical images were processed into a 3D FEM model of the human head for this investigation. In the literature, there are now many open- and closed-source databases from which to extract these types of medical raw data. The specific model considered in this study is derived from the NY Head Model constructed by the Parra Lab group at the City University of New York [28]. The segmentation data were obtained by averaging three different MRI sources for various tissues: the brain is acquired from the symmetric ICBM-152 v2009, non-brain tissues are extrapolated from the symmetric ICBM-152 v6, and the lower portion of the head from [28]. The general model was based and validated on four individual heads, whereby a precise FEM model was built for each of them [29]. The final segmentation files after the averaging operation involve the symmetric geometry of the entire head. This was taken into consideration due to the symmetric properties exhibited by objects imported into COMSOL Multiphysics, which can significantly reduce the computational cost of any simulations.

The provided dataset from [28] was processed and tissue regions for the skin, skull, and brain were extracted. To process the *.nifti* segmentation files, two software programs were used: *MATLAB* R2023b for creating closed and volumetric 3D objects, and *3D Slicer* 5.2.2 for refining geometry operations. In particular, the *iso2mesh* 3D library in *MATLAB*, which provides excellent computational capabilities for binary and grayscale volumetric images such as segmented MRI/CT scans, was utilized. 

The *.nifti* files for each of the three tissues were loaded, opened, and converted into binary logical values to identify each grayscale level of the segmented geometry. Subsequently, a closing operation was performed on each geometry to obtain a final closed water-tight object, thus avoiding “holes” that could introduce discontinuities in the final mesh. Utilizing the *fillholes3d.m* function with a gap size of 55 for each of the three tissues resulted in the creation of the three objects. The gap size is a crucial parameter, representing the size of the hole to be filled in the geometry. In this case, a trade-off was sought, as large gap sizes would lead to a geometry significantly different from the original, especially for the brain, while very small values would introduce an almost negligible approximation in the geometry.

Subsequently, the 3D binary image was converted into an actual volume using the *imedge3d.m* function which extracts contour voxels from a binary image. Finally, the object was reconverted into the *.nifti* format and exported.

For the final processing, *3D Slicer* was used. The three objects were imported into the software after being processed in *MATLAB*. Due to the reduced complexity of the geometry after the previous closing operation, the 3D representation of the object was obtained using the Otsu thresholding method. Subsequently, cutting operations were performed in *3D Slicer*, allowing for the manual removal of small volumes from the object. These volumes are considered undesirable for the final purpose, as they would provide extra material for meshing without a specific purpose. Additionally, the lower part of the skull (including the first vertebrae of the spine) was partially removed, as this part of the head is negligible for the simulations.

After the cutting operation, some of the modified surfaces underwent shape changes. To address this, classic morphological opening and closing operations were applied to remove small extrusions remaining in the geometry and fill small residual holes. Finally, a smoothing operation was performed; more specifically, Gaussian smoothing was used.

**Figure 2:**
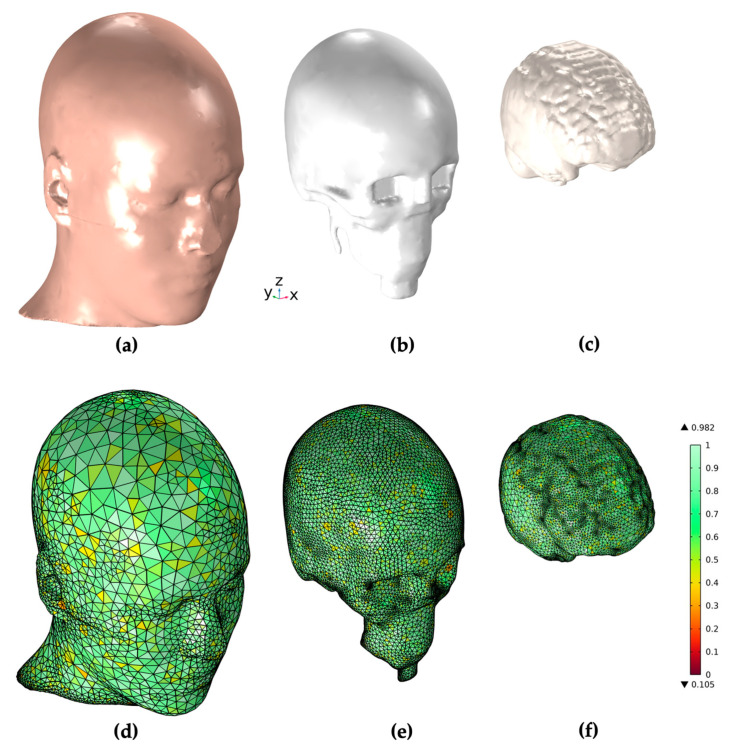
COMSOL Multiphysics geometry and mesh generation of the three tissue regions of the head. (**a**–**c**) show the geometry of the skin, skull, and white matter region, while (**d**–**f**) illustrate the meshed geometries for those regions in the stand-alone head model.

Moreover, only for the skull, a “grow” operation was carried out using the *margin operation* tool to give it a thickness of about 3 mm. The uniform thickness of the skull adds a high degree of homogeneity to the final mesh but represents a strong approximation of the skull, while the real thickness of the skull is not uniform along the skullcap. The last step in 3D Slicer was to export the geometry in *.stl* format, ready to be imported into COMSOL Multiphysics 6.1. Figure 2 illustrates the resulting geometry and mesh of the three tissue regions of the head.

In order to perform simulations including the electromagnetic properties of the human head, each region was assigned a specific conductivity and relative permittivity value taken from the literature [30,31,32]. Table 1 gives an overview of the utilized values.

### 2.2. ME Sensor Model

**Figure 3:**
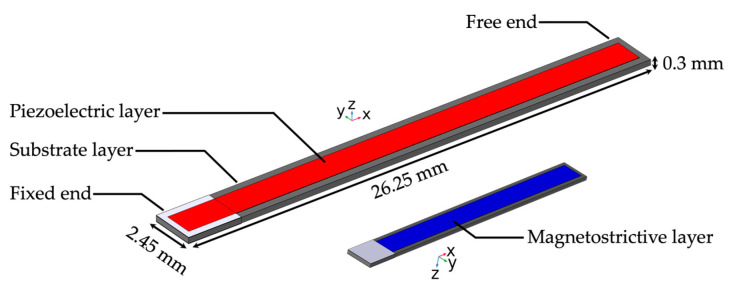
The ME sensor model. The cantilever sensor is shown with substrate layer in grey and the piezoelectric layer on the surface in red. The left end of the sensor is clamped, while the right end is free, resulting in the fixed-free bending mode operation. The smaller inset shows the opposite side of the substrate with the magnetostrictive layer on top in blue. The length, width, and thickness for the substrate layer are given in mm.

We designed and implemented the ME sensor based on the ME sensor models from our previous work [26,27]. Each sensor consisted of a substrate layer of silicon with 26.25 mm length, 2.45 mm width, and 300 µm thickness, a magnetostrictive layer of FeCoSiB with 22.90 mm length, 1.80 mm width, and 20 µm thickness, and a piezoelectric layer of aluminum nitride (AlN) with 25.60 mm length, 1.60 mm width, and 20 µm thickness. The AlN is polycrystalline, and both the magnetostrictive and piezoelectric layer can be produced for experimental measurements via an in-house magnetron sputtering process [33]. The magnetostrictive and piezoelectric layers were located on the opposite sides of the substrate layer. The thickness of the active layers was chosen at a factor of 10 times higher than that typically used in experimental sensors at Kiel University to reduce the computation time. The sensor operated in resonant bending mode and fixed-free configuration at a resonance frequency of 848 Hz. Figure 3 gives an overview of the ME sensor geometry.

Table 2 gives an overview of the three layers forming the composite ME sensor. The magnetostrictive layer, piezoelectric layer, and substrate layer each have their unique material parameters which are given in Appendix A. A linearized material model was used at the sensor’s small-signal working point. Details of the applied boundary conditions and physical properties of the three layers are described in our previous work [26,27].

The mathematical model and physical properties of cantilever ME sensors consisting of ideal and slipless composite layers are governed by systems of differential and constitutive equations which are characterized in detail in previous studies [30,34,35,36,37,38]. The equations that we discuss below define our 3D-FEM model and are utilized in three built-in physics interfaces in COMSOL Multiphysics 6.1: the *solid mechanics*, *magnetic fields*, and *electrostatics* interfaces. This set of equations was also utilized and described in our previous work containing ME sensors and a simplified human head model [12]. It is repeated here for easy access. Beginning with *solid mechanics*, this interface describes a set of equations that couples the mechanical, electrical, and magnetic properties of the ME sensor and contains specific terms for each layer.

(1) $$-\rho \omega^{2}u=\nabla \cdot S$$

(2) S=C :ε

(3) $$\varepsilon =\frac{1}{2}\left[{\left(\nabla u\right)}^{T}+\nabla u\right]$$

(4) C=C(E,ν).

The first set of Equations (1)–(4) corresponds to the *linear elastic material* node which covers the general mechanical properties of the model and includes S and ε for stress and strain, u as the displacement vector, the density ρ and the angular frequency ω. For the silicon substrate specifically, the coupling between stress and strain is a function of its Young’s modulus and Poisson’s ratio and is described by C in Equation (2).

The magnetostrictive and piezoelectric layers have separate physics nodes discussed below, starting with the *magnetostrictive material* node:

(5) $$S=c_{H}\;:\varepsilon -H\cdot e_{HS}$$

(6) $$B=\mu_{0\;}\mu_{rS}H+e_{HS}\;:\varepsilon .$$

Equations (5) and (6) include the coupling matrix in Voigt notation $e_{HS}$ and the elasticity matrix $c_{H}$. This node also governs the relation between the magnetic flux density vector B, the magnetic field vector H, and the relative permeability $\mu_{rS}$ of the material. Analogous to the *magnetostrictive material* node, the *piezoelectric material* node has a specific set of equations that describe its properties, including the coupling of the ME sensor’s electric and elastic properties:

(7) $$\nabla \cdot D=\rho_{v}$$

(8) $$S=c_{E}\;:\varepsilon -E\cdot e_{ES}$$

(9) $$D=\varepsilon_{0\;}\varepsilon_{rS}E+e_{ES}\;:\varepsilon .$$

In Equation (7), Gauss’s law for the relation between the electric displacement field D and the volume charge density is applied to the model. Equations (8) and (9) include the coupling matrix in Voigt notation $e_{ES}$ and the elasticity matrix $c_{E}$, as well as E for the electric field vector and $\varepsilon_{rS}$ for the relative permittivity. We used the stress-magnetization and the stress-charge form for the magnetostrictive and piezoelectric material nodes, respectively. The Equations (1)–(9) that we described so far are applied in the *solid mechanics* physics node of our model and include elastic material properties, as well as coupling between the sensor layers. The electric and magnetic behavior of our model is governed in the *magnetic fields* and *electrostatics physics* nodes of our model given in Equations (10)–(12):

(10) ∇×H=σE+jωD

(11) B=∇×A

(12) E=−∇V−jωA

(13) $$D=\varepsilon_{0\;}\varepsilon_{r}E$$

(14) $$B=\mu_{0\;}\mu_{r}H.$$

With Equations (10)–(12) we utilized Maxwell’s equation and related electric and magnetic fields to the magnetic vector potential A. The equation for the electric field strength used in the model is given in (12). Equations (13) and (14) apply via the boundary condition *Ampère’s law* in the *magnetic fields* physics interface to the silicon substrate. Additionally, this boundary condition defines the general constitutive relations of ***D*** and ***E*** for the magnetostrictive material, as well as ***B*** and ***H*** for the piezoelectric material, in instances where no more specific physical properties are assigned, i.e., no magnetostriction for the piezoelectric material or piezoelectricity for the magnetostrictive material. With the set of equations given in (1)–(14), our multiscale combined ME sensor and human head model can be simulated with different electric and magnetic excitation methods, and full magnetoelectric coupling of the sensor layers.

### 2.3. ME Sensor Array Model

**Figure 4:**
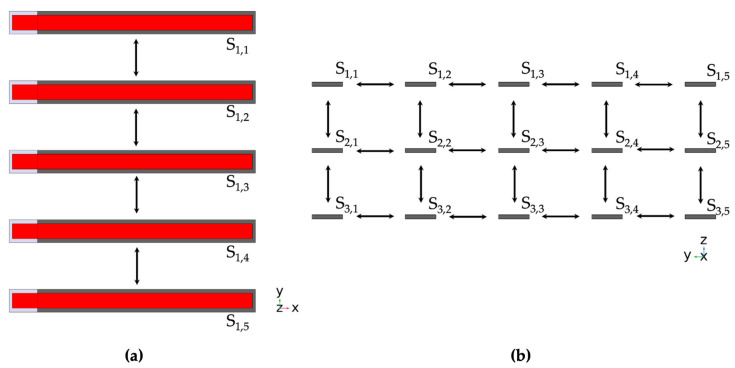
(**a**) Top-down view and (**b**) frontal view (on fixed end) of the 15-sensor array. Between each adjacent sensor is a variable distance between 1 mm and 5 cm. This figure shows equidistant sensor placement with 5 mm between the neighbors in both vertical and horizontal direction. The variable distance allowed us to investigate the magnetic crosstalk between sensors and analyze the influence of the magnetostrictive layers on nearby ME sensors.

The aim of this work is to combine 15 individual ME sensors into a sensor array and investigate the response of each individual sensor based on its relative position in the array and distance from the neighboring sensors for different types of magnetic excitation. To achieve this goal, we organized the sensors into a 3 × 5 grid to form an array with three rows and five columns of adjacent sensors. The long axis of the sensors was parallel to the *x*-axis and the piezoelectric layer faced upwards in *z*-direction. The distances between the sensors in the array varied from 5 mm up to 5 cm in the vertical and horizontal directions simultaneously. Figure 4 illustrates the array configuration with the distance between neighboring sensors set to 5 mm. An array of these or similar dimensions was chosen due to in-house fabrication approaches at Kiel University and the possibility of using the array in an operation room, as a smaller array with variable positioning would potentially allow surgical procedures without covering the entire head.

### 2.4. Combined MRI-Based Human Head and ME Sensor Array Model

After establishing the human head and ME sensor array models separately, we combined both parts into the joint multiscale model. The sensors in the array can be located at arbitrary positions in space and distances both from the head and adjacent sensors. The challenge to be overcome was to combine both the anatomical head model, as well as the ME sensor array model with 15 sensors, into one combined multiscale and multiphysics model. A core consideration for this was the differences in dimensions between the different model domains. While the sensors’ PE and MS layers had a thickness of only 20 µm, the diameter of the head was approximately 20 cm. This translated to a vastly different size and resolution of mesh elements for the differently sized domains, because the mesh is designed such that extremely thin layers are of a sufficiently small size, while larger domains are modeled with larges element sizes to reduce the computational load. The number of degrees of freedom (DOF) solved for in a combined model with three ME sensors demonstrated in previous work [27] is approximately 25 million, while the number increases to approximately 70 million DOF when including 15 ME sensors in the array. The number of DOF is determined by the amount of mesh elements, as well as the utilized physics in the model, and is an important figure of merit for the computational requirements to solve a given FEM model. Details on the mesh parameters for all structures in a model with three ME sensors are given in Appendix B, with sizes referring to the longest edge of tetrahedral mesh elements. 

Increasing the number of sensors from three to 15 posed a challenge in terms of computation time and hardware requirements for the computer running our simulations, since the number of DOF almost tripled in the combined model with a larger array and full mechanical, magnetic and electric coupling. Using the mesh parameters given in Appendix B, we were not able to achieve a successful simulation run with a converged solution. In order to achieve a solution, we iteratively adjusted some parts of the mesh to be coarser than in the previous study [27], while keeping the mesh resolution as high as possible for the sensor geometry. The adjusted mesh parameters for this investigation step included the head geometry, the air environment, and the substrate layer of the ME sensors. The magnetostrictive and piezoelectric layers remained unchanged. The mesh parameters for the combined model with 15 sensors are given in Appendix C. Inspecting the mesh yielded some elements of low quality for the figure of merit skewness, but no significant number of elements of poor quality (defined by skewness under 0.1 according to COMSOL’s guidelines). The coarser mesh might have adversely impacted the accuracy of the solutions provided in the results section, but was necessary to enable our simulations to finish successfully.

Due to challenges with the numerical stability of our solutions, two simplifying conditions had to be applied to our model. Firstly, based on communications with COMSOL employees, calculating 3D FEM models with very high differences in material parameters such as the specific conductivity may result in a failure to find a converged solution [39]. In this case, this affects the near-zero specific conductivity of air which fills most of the modelling space. A recommended solution for this is to artificially increase the specific conductivity of the material in question sufficiently; thus, we set the conductivity of our air domain to 1 × 10^−6^ S/m. Secondly, we increased the numerical stability of our simulations with dipole excitation by positioning a second dipole with the same orientation and a magnitude 1 µA·m at a distance of 2 mm directly below the main excitation dipole of magnitude 1 mA·m. Due to the factor of a thousand between dipole magnitudes, the contribution of the secondary dipole to the overall electromagnetic field is considered negligible, while empirically improving the convergence of the utilized indirect solver we used. The application of similar conditions to improve numerical stability was also discussed in our previous work [27].

## 3. Results

The results in this section are categorized into two different types of magnetic field excitation. First, we looked at the array’s response in a constant, homogeneous magnetic field, which is applied to the entire model volume. Following that, we replaced the homogeneous magnetic field excitation with a single electric current point dipole source inside the white matter region of the head model. For the investigation of the crosstalk effect, the distance between adjacent sensors in both the horizontal and vertical directions was varied in four steps within an interval between 5 mm and 5 cm. As the distance between neighboring sensors changes, the magnetic flux between them changes direction and is guided inside the highly permeable magnetostrictive layers of the sensors. For each sensor distance, a simulation with either homogeneous or dipole excitation was performed and the response for each sensor in the array was evaluated. Different distances between sensors result in different sensor responses depending on the position inside the array, as the following sections will demonstrate.

### 3.1. Homogeneous Excitation

**Figure 5:**
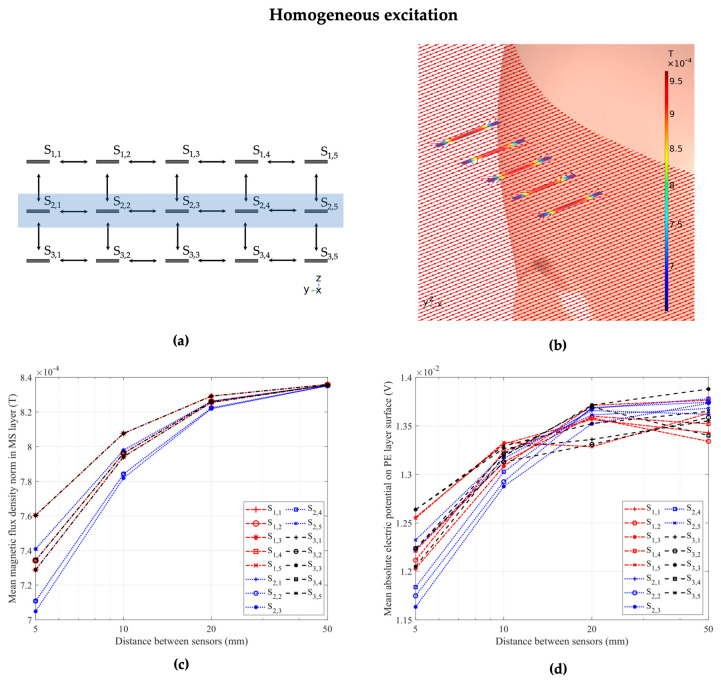
(**a**) Schematic of the 15-sensor array in top-down and frontal view. The blue rectangle marks the second row of five sensors, which we used to visualize the crosstalk effects between sensors. The distance between sensors in this schematic is 5 mm. (**b**) The 15-sensor array in a homogeneous magnetic field. The magnetic field strength is 1 A/m and is applied in *x*-direction. The highlighted middle row of five sensors within the array, namely the sensors S_2,1_, S_2,2_, S_2,3_, S_2,4_, and S_2,5_, is shown, with clear crosstalk effects between sensors. The distance between the sensors is 1 cm in this exemplary position. (**c**) The magnetic flux density inside the MS layers and (**d**) the electric potential on the surface of the PE layer of each of the 15 sensors of the array at different distances from adjacent sensors, showcasing crosstalk at small distances between the sensors and increased effects in the central sensors.

We performed magnetic excitation of the ME sensor array using a homogeneous magnetic field strength of 1 A/m in *x*-direction applied to the entire model environment. The aim of this study step was to establish and validate the basic sensor response to a simple excitation field and investigate potential crosstalk effects independently of influences of inhomogeneous field effects. Before each array simulation, validation steps were conducted for homogeneous and dipole excitation, i.e., the sensor material parameters were set to those of the air environment in order to eliminate geometric or numeric inconsistencies and validate the magnetic flux density inside the model domain without the presence of high-permeability sensor material. Figure 5a shows a schematic of the full array and highlights the five sensors of the middle row, namely sensors S_2,1_, S_2,2_, S_2,3_, S_2,4_, and S_2,5_. We further highlight the exemplary behavior of these five sensors in Figure 5b, where we display the sensors in a homogeneous excitation magnetic field in *x*-direction and the corresponding magnetic flux density inside the magnetostrictive layer for each sensor. The distance between adjacent sensors was 1 cm. The outermost sensors S_2,1_ and S_2,5_ exhibited the highest magnetic flux density, followed by sensors S_2,2_ and S_2,4_. The central sensor, S_2,3_, exhibited the lowest magnetic flux density out of all sensors in the array due to its central position and the resulting crosstalk with its adjacent sensors. Finally, Figure 5c gives a plot of the magnetic flux density norm inside every sensor of the full array with 15 sensors for different distances between adjacent sensors. The previously observed behavior of high crosstalk between adjacent sensors at low distances is clearly visible, while the closer a sensor is to the center of the array, the stronger the effect. At a distance of 5 cm between the sensors, the crosstalk effect is negligible, and all sensors exhibit approximately the same response. At each individual distance between adjacent sensors, the sensor with the lowest magnetic flux density was the middle sensor (S_2,3_), while the highest flux density was shared between the four corner sensors (S_1,1_, S_1,5_, S_3,1_, S_3,5_). At the lowest distance of 5 mm, the flux density in the corner sensors was 7.9% higher than in the middle sensor. For the central sensor (S_2,3_), the magnetic flux density was 15.6% lower at a distance of 5 mm compared to a distance of 5 cm to its neighbors. Notably, the crosstalk effect was significantly stronger with up to 11% for sensors with closely vertically adjacent sensors, compared to horizontally adjacent sensors, based on simulations considering exclusively horizontally or vertically adjacent sensors. For the electric potential, Figure 5d offers similar general behavior for the crosstalk effect based on the electric behavior. Here, while the values converge for all sensors at a distance of 5 cm between neighbors for the magnetic flux density, the electric potential still sees a difference of approximately 4% between the highest (S_33_) and lowest (S_12_) at that distance. We expect numerical error to this degree based on the calculation of the fully coupled magnetoelectric effect between the layers as a possibility for the slightly diverging behavior of the electric response of the sensors.

### 3.2. Dipole Excitation

After evaluation of the behavior of the ME sensor array in homogeneous magnetic field excitation, we moved towards a specific inhomogeneous excitation mechanism. An electric current point dipole was placed at coordinates *x* = 30 mm, *y* = −20 mm, and *z* = 30 mm inside the white matter compartment of the head geometry, within the approximated right cerebral hemisphere. This configuration serves as a representative of a deep-brain stimulation scenario. The dipole moment, combined with the electric properties of the head, results in an induced magnetic field propagating through the head and the air environment into the ME sensor array, enabling us to evaluate its response and gain further insights into the behavior of the individual sensors in inhomogeneous excitation.

**Figure 6:**
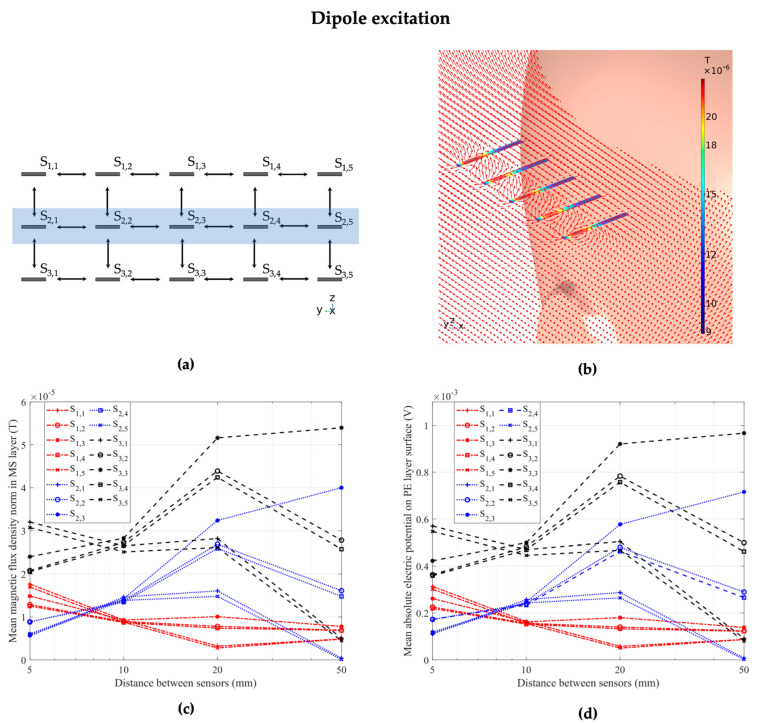
(**a**) Schematic of the 15-sensor array in top-down and frontal view. The blue rectangle marks the second row of five sensors, which we used to visualize the crosstalk effects between sensors. The distance between sensors in this schematic is 5 mm. (**b**) The 15-sensor array in a dipole magnetic field. The dipole moment is 1 mA·m in magnitude and oriented in y-direction. The highlighted middle row of five sensors within the array, namely the sensors S_2,1_, S_2,2_, S_2,3_, S_2,4_, and S_2,5_, is shown, with possible crosstalk and flux concentration effects between sensors. The distance between the sensors is 1 cm in these exemplary positions. (**c**) The magnetic flux density inside the MS layers and (**d**) the electric potential on the surface of the PE layer of each of the 15 sensors of the array at different distances from adjacent sensors, showcasing an increased sensor response in the bottom row of sensors in the array (S_3,1_–S_3,5_), but inconsistent behavior with increasing sensor distance.

Based on the orientation of the dipole and the ME sensor, the sensor response can be vastly different due to its directional sensitivity, as we showed in our previous work with an array of orthogonally oriented ME sensors and different dipole orientations [27]. For this work, we exemplarily investigated only one dipole orientation (*y*-direction) and one sensor orientation for each sensor in the array (*x*-direction), but modifications to this model with arbitrary configurations for arrays and sources are possible for further analysis. The dipole can be configured with an arbitrary dipole moment direction, amplitude, and location in the head. We chose an exemplary dipole moment of 1 mA·m in *y*-direction. The chosen dipole amplitude is in agreement with studies on deep brain stimulation (DBS) and head models from the literature [32,40]. As seen in previous investigations, it is not trivial to predict the behavior of the sensor array when excited by an inhomogeneous magnetic field. As seen in Figure 6, the individual sensors’ magnetic and electric behavior does not follow specific patterns with increasing distance to neighbors. Some indicators can partly predict the behavior; for example, the fact that in the vertical array configuration, the bottom row of sensors exhibits a higher magnetic flux density based on proximity to the human head and the magnetic field propagation through the tissue. In this case, all five sensors in the bottom row of sensors (S_3,1_–S_3,5_) exhibited the highest magnetic and electric response at distances of between 5 mm and 1 cm between adjacent sensors. Similarly, four out of the five sensors (S_3,1_–S_3,4_) and three out of the four sensors (S_3,2_–S_3,4_) with the highest magnetic flux density and electric potential at distances of 2 cm and 5 cm from their neighbors, respectively, are sensors from the bottom row of the array. 

## 4. Discussion

**Figure 7:**
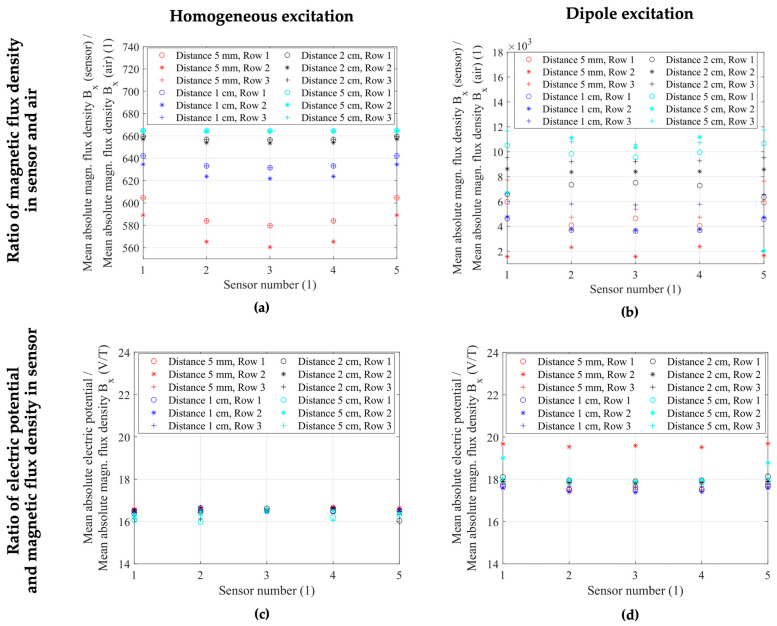
(**a**,**b**)The ratio between the mean absolute x-component of the magnetic flux density inside the MS layer geometry for sensor material parameters and sensor material parameters set to air in (**a**) homogeneous and (**b**) dipole excitation cases. This ratio is a measure of the field concentration and depends on the sensor position in the array. (**c**,**d**) The ratio between the mean absolute electric potential over the PE layer and the mean absolute magnetic flux density component in *x*-direction in each sensor of the array and for different distances between adjacent sensors. (**c**) illustrates the near-constant factors between 16.0 and 16.6 for the ratio for each sensor and each position within the array in homogeneous excitation. (**d**) shows a factor between 17.4 and 18.0 for most sensors, with the outlying second row of sensors (S_2,1_–S_2,5_) at the minimum distance of 5 mm between adjacent sensors exhibiting a factor between 19.5 and 19.7.

Magnetic crosstalk effects between the adjacent ME sensors are clearly observed for the homogeneous excitation case in Figure 5; they are more challenging to visualize for the inhomogeneous case in Figure 6 with a strong spatial variation of the magnetic field strength and direction. To gain a better understanding of the observed results, we investigated the sensor sensitivity. As the ME sensor has a previously demonstrated directional sensitivity [27], we investigated the effect of the *x*-component of the magnetic field, which is parallel to the cantilever’s long axis. We calculated two ratios between important model parameters in order to discuss the presented sensor behavior in both homogeneous and inhomogeneous excitation cases. The first ratio was between the mean absolute B*_x_* component of the magnetic flux density inside the MS layer of the sensor, and the same layer with its material parameters set to those of air. The second ratio was between the mean absolute electric potential as the electric sensor response and the B*_x_* component of the magnetic flux density inside the magnetostrictive layer of each sensor. Figure 7a,b demonstrate the results for the ratios between sensor and air magnetic flux densities, while Figure 7c,d visualize the ratios between electric potential and sensor magnetic flux density for both excitation cases and each individual sensor inside the array. Here, the horizontal axis determines the sensor number with horizontally adjacent sensor columns (S_1,n_–S_1,n_), while the markers differentiate between the vertically adjacent rows of sensors (S_m,1_–S_m,3_), as illustrated in Figure 5a and Figure 6a. The four different colors represent the different distances between adjacent sensors, with distances of 5 mm, 1 cm, 2 cm, and 5 cm in the horizontal and vertical directions, respectively. The results show that, while we observe magnetic flux densities and piezoelectric voltages that are highly dependent on the sensor position and distance to its neighbors in both homogeneous and inhomogeneous excitation, the ratio between electric response and excitation field was near constant for each excitation case. 

As seen in Figure 7a,b, the ratio between the magnetic flux densities inside the sensor geometry and the corresponding air volumes were not constant with up to two orders of magnitude in the homogeneous and four orders of magnitude in the inhomogeneous case between the ratios at different sensor positions. This large spread showcases the field concentration effect of the magnetostrictive material and highly position-dependent behavior of the sensors. Contrary to the highly variable ratios between the magnetic flux densities, Figure 7c illustrates a narrow range of values between 16.0 and 16.6 for the ratio between electric potential and magnetic flux density for the homogeneous excitation case and any sensor at the investigated array positions and distances. For the inhomogeneous case, Figure 7d shows different behavior between the middle row of sensors (Row 2, S_2,1_–S_2,5_) at a distance of 5 mm between adjacent sensors and every other configuration. A factor between 17.4 and 18.0 was calculated for every sensor position, with outliers for the first and last sensor in the second row (S_2,1_ and S_2,5_) at a distance of 5 cm exhibiting a factor of approximately 19.0. 

The entire second row of sensors (S_2,1_–S_2,5_) at the minimum distance of 5 mm between sensors exhibited a factor between 19.5 and 19.7. Despite the outlying row of sensors in the inhomogeneous case, all 15 sensors in either homogeneous or dipole excitation fields exhibited similar, near-constant ratios between the electric response and the magnetic excitation. Considering the clear crosstalk effects demonstrated for the homogeneous case in Figure 5 and the seemingly inconsistent behavior in dipole excitation seen in Figure 6, with up to two orders of magnitude difference in magnetic flux density between different sensor positions in Figure 6c, these near-constant ratios demonstrated a highly linear relation between the excitation magnetic field and the electric sensor response, including potential crosstalk and flux concentration effects. Therefore, the change in the electric sensor response may be attributed to the change in the magnetic field in the sensor.

## 5. Conclusions

We have shown a combined ME sensor array and MRI-based human head model for joint biomagnetic field simulations and analysis of ME sensor behavior. The combined model allowed us to place an electric dipole source inside the head and simulate the excitation and propagation of an electromagnetic field through the head’s tissue regions, the air environment, and into the array of ME sensors. As an alternative study step to the dipole excitation, a homogeneous excitation field was also applied to the entire simulation environment and the sensor responses were evaluated.

The results that we presented offer insights on the response of individual ME sensors within an array configuration to different excitation mechanisms. For an array of 15 ME sensors in a homogeneous magnetic excitation field, a magnetic crosstalk effect between adjacent sensors is clearly visible in Figure 5. The magnitude of this effect decreases with increasing distance between adjacent sensors and becomes negligible at a distance of 5 cm. In the case of inhomogeneous excitation, a strong change in the sensor response is obtained as seen in Figure 6 due to the position-dependent magnetic field strength and direction. The sensor response, in this case, depends on the position and orientation of the array relative to the source inside the head, as well as the propagation of the electromagnetic field through the head based on the electric tissue properties and geometry of the head [27]. In excitation fields that have large vector components in directions other than the sensitive direction of the ME sensor, differences between sensor positions may be more pronounced, as opposed to a homogeneous excitation field in a sensitive direction, which is demonstrated in this study. Another effect that has to be considered is the inverse magnetostrictive effect, which is caused by strain in the magnetostrictive layer and can lead to superimposing local fields on top of the excitation field [34]. This effect could have a varying degree of influence on the behavior of adjacent sensors, particularly in inhomogeneous magnetic fields.

To investigate whether the linear relation between the excitation magnetic field and the electric sensor response is maintained, we calculated the ratio of these quantities for each individual ME sensor in both excitation setups and four different distances between adjacent sensors inside of the array. We demonstrated that even with large differences up to factors of two orders of magnitude in the magnetic and electric sensor responses between sensors in various combinations of array geometry and magnetic field sources, the ratio between the electric potential and magnetic flux density inside the sensors was near-constant in homogeneous (16.0–16.6) and inhomogeneous (17.4–19.7) excitation. This result is shown in Figure 7 and confirms that the behavior of each ME sensor was linear as expected for the linear model, even though a highly position-dependent field concentration is demonstrated. Therefore, for the linear approximation, a separation of the excitation source and sensor model in both homogeneous and inhomogeneous excitation configurations is possible. In the next step, the FEM model can be extended to investigate nonlinear effects due to nonlinear material properties as well as effects due to nonlinear secondary currents induced inside the head.

In summary, this work contains insights into the response of ME sensors within different array configurations for homogeneous and inhomogeneous dipole magnetic field excitation for the small-signal linearized case. The sensor response strongly depends on the excitation vector field and confirms the influence of magnetic crosstalk between sensors. Further research may include additional simulations with different array configurations in inhomogeneous fields to evaluate the near-constant sensitivity of the sensors. Additionally, results could be compared between separately evaluated source and sensor simulations and the sensor response in combined models such as the one presented in this work. Future excitation models could also evolve the head geometry demonstrated in this work to contain more tissue regions in higher resolution, as well as different biological or artificial excitation sources such as implanted DBS electrodes, giving rise to potential localization and orientation investigations with patient- or application-specific head and ME sensor models.

## Figures and Tables

**Table 1:** The regions of the human head model with their respective specific conductivity and relative permittivity taken from literature.

Region	Specific Conductivity [30,31]	Relative Permittivity [32]
Skin	1.00 S/m	1,200,000
Skull	0.05 S/m	40,000–1,000,000
White Matter	0.43 S/m	30,000,000

**Table 2:** The layers of the ME sensor model with their materials, length, width, and height.

Layer	Material	Length	Width	Height
Magnetostrictive	FeCoSiB	22.90 mm	1.80 mm	20.00 µm
Piezoelectric	AlN	25.60 mm	1.60 mm	20.00 µm
Substrate	Si	26.25 mm	2.45 mm	300.00 µm

## Data Availability

The data that the findings of this work are based on are available from the corresponding authors upon reasonable request.

## Appendix A. Material Parameters

The material parameters of AlN, FeCoSiB, and silicon at the working point used for the ME sensor are based on literature and in-house characterization and are given in this section [35,41,42,43].

AlN:

(A1) $$c_{E,\mathrm{AlN}}=\left(\begin{array}{cccccc} 41 & 14.9 & 9.9 & 0 & 0 & 0\\ 14.9 & 41 & 9.9 & 0 & 0 & 0\\ 9.9 & 9.9 & 38.9 & 0 & 0 & 0\\ 0 & 0 & 0 & 12.5 & 0 & 0\\ 0 & 0 & 0 & 0 & 12.5 & 0\\ 0 & 0 & 0 & 0 & 0 & 12.5 \end{array}\right)\times 10^{10}\;\mathrm{Pa}$$

(A2) $$e_{ES,\mathrm{AlN}}=\left(\begin{array}{cccccc} 0 & 0 & 0 & 0 & -0.48 & 0\\ 0 & 0 & 0 & -0.48 & 0 & 0\\ 9.9 & 9.9 & 38.9 & 0 & 0 & 0\\ -0.58 & -0.58 & 1.55 & 0 & 0 & 0 \end{array}\right)\times C/m^{2}$$

(A3) $$\rho_{\mathrm{AlN}}=3300\;\mathrm{kg}/m^{3}$$

(A4) $$\varepsilon_{\mathrm{AlN}}=80\times 10^{-12}\;F/m$$

(A5) $$\mu_{\mathrm{AlN}}=0.4\pi \times 10^{-6}\;H/m$$

FeCoSiB:

(A6) $$c_{H,\mathrm{FeCoSiB}}=\left(\begin{array}{cccccc} 150 & 45 & 45 & 0 & 0 & 0\\ 45 & 150 & 45 & 0 & 0 & 0\\ 45 & 45 & 150 & 0 & 0 & 0\\ 0 & 0 & 0 & 40 & 0 & 0\\ 0 & 0 & 0 & 0 & 40 & 0\\ 0 & 0 & 0 & 0 & 0 & 40 \end{array}\right)\times 10^{10}\;\mathrm{Pa}$$

(A7) $$e_{HS,\mathrm{FeCoSiB}}=\left(\begin{array}{cccccc} 8500 & -2833.3 & -2833.3 & 0 & 0 & 0\\ 0 & 0 & 0 & 0 & 0 & 0\\ 0 & 0 & 0 & 0 & 0 & 0\\ 0 & 0 & 0 & 0 & 0 & 0 \end{array}\right)\times N/\left(\mathrm{Am}\right)$$

(A8) $$\rho_{\mathrm{FeCoSiB}}=7250\;\mathrm{kg}/m^{3}$$

(A9) $$\varepsilon_{\mathrm{FeCoSiB}}=8.85\times 10^{-12}\;F/m$$

(A10) $$\mu_{\mathrm{FeCoSiB}}=1.13\times 10^{-3}\;H/m$$

Silicon:

(A11) $$E_{\mathrm{Si}}=170\times 10^{9}\;\mathrm{Pa}$$

(A12) $$\nu_{\mathrm{Si}}=0.28$$

(A13) $$\rho_{\mathrm{Si}}=2329\;\mathrm{kg}/m^{3}$$

(A14) $$\varepsilon_{\mathrm{Si}}=103.59\times 10^{-12}\;F/m$$

(A15) $$\mu_{\mathrm{Si}}=0.4\pi \times 10^{-6}\;H/m$$

## Appendix B. Mesh Parameters for Original 3-Sensor Array Model

Head and sensor model geometry parameters for a model with 3 ME sensors [27]. The mesh is designed so that extremely thin layers of PE and MS material are of a sufficiently small size, while larger structures such as the head and airbox are allowed larger element sizes to decrease the total number of degrees of freedom and therefore the computational load for the simulations. Values given are for the longest edges of tetrahedral mesh elements.

**Mesh Parameter**	**PE and MS Layer**	**Substrate Layer**	**Skin**	**Skull**	**White Matter**	**Airbox**
Max. element size	5.00 × 10^−4^ m	1.00 × 10^−3^ m	5.25 × 10^−2^ m	5.25 × 10^−2^ m	5.25 × 10^−2^ m	3.61 × 10^−2^ m
Min. element size	9.00 × 10^−5^ m	1.00 × 10^−4^ m	1.00 × 10^−3^ m	1.00 × 10^−3^ m	1.00 × 10^−3^ m	1.00 × 10^−3^ m
Max. element growth rate	3	3	1.45	1.45	1.45	1.4
Curvature factor	0.3	0.3	0.5	0.5	0.5	0.4
Resolution of narrow regions	3	3	0.6	0.6	0.6	0.7

## Appendix C. Mesh Parameters for Modified 15-Sensor Array Model

In order to be able to run simulations with the computationally highly demanding 15-sensor array model on our hardware, modifications to the mesh parameters given in Appendix B had to be made. The following table includes the modified minimum element sizes for the substrate layer, the head geometry, and the surrounding air volume. The sensors’ piezoelectric and magnetostrictive layers remain unchanged.

**Mesh Parameter**	**PE and MS Layer**	**Substrate Layer**	**Skin**	**Skull**	**White Matter**	**Airbox**
Max. element size	5.00 × 10^−4^ m	1.00 × 10^−3^ m	5.25 × 10^−2^ m	5.25 × 10^−2^ m	5.25 × 10^−2^ m	3.61 × 10^−2^ m
Min. element size	9.00 × 10^−5^ m	3.00 × 10^−4^ m	6.56 × 10^−3^ m	6.56 × 10^−3^ m	6.56 × 10^−3^ m	2.63 × 10^−3^ m
Max. element growth rate	3	3	1.45	1.45	1.45	1.4
Curvature factor	0.3	0.3	0.5	0.5	0.5	0.4
Resolution of narrow regions	3	3	0.6	0.6	0.6	0.7
